# First international consensus on the methodology of lymphangiogenesis quantification in solid human tumours

**DOI:** 10.1038/sj.bjc.6603445

**Published:** 2006-11-21

**Authors:** I Van der Auwera, Y Cao, J C Tille, M S Pepper, D G Jackson, S B Fox, A L Harris, L Y Dirix, P B Vermeulen

**Affiliations:** 1 Translational Cancer Research Group Antwerp, Laboratory of Pathology, University of Antwerp/University Hospital Antwerp, Edegem 2650, Belgium; Oncology Centre, General Hospital Sint-Augustinus, Wilrijk 2610, Belgium; 2 Laboratory of Angiogenesis Research, Microbiology and Tumor Biology Center, Karolinska Institutet, Stockholm 171 77, Sweden; 3 Department of Microbiology, Laboratory of Angiogenesis Research, Tumor and Cell Biology, Karolinska Institutet, Stockholm 171 77, Sweden; 4 NetCare Molecular Medicine Institute, Unitas Hospital and Department of Immunology, Faculty of Health Sciences, University of Pretoria, Pretoria 0002, South Africa; 5 Medical Research Council Human Immunology Unit, Weatherall Institute of Molecular Medicine, John Radcliffe Hospital, Oxford OX3 9DS, UK; 6 Department of Pathology, Peter MacCallum Cancer Centre, Victoria 8006, Australia; 7 Cancer Research UK Molecular Oncology Laboratories, Weatherall Institute of Molecular Medicine, University of Oxford, John Radcliffe Hospital, Oxford OX3 9DS, UK

**Keywords:** lymphangiogenesis, lymphatic vessel density (LVD), lymphatic endothelial cell proliferation (LECP), podoplanin, lymph node metastasis, vascular endothelial growth factor (VEGF)

## Abstract

The lymphatic system is the primary pathway of metastasis for most human cancers. Recent research efforts in studying lymphangiogenesis have suggested the existence of a relationship between lymphatic vessel density and patient survival. However, current methodology of lymphangiogenesis quantification is still characterised by high intra- and interobserver variability. For the amount of lymphatic vessels in a tumour to be a clinically useful parameter, a reliable quantification technique needs to be developed. With this consensus report, we therefore would like to initiate discussion on the standardisation of the immunohistochemical method for lymphangiogenesis assessment.

Metastasis is the leading cause of cancer mortality. Metastatic cancer cells can escape from their site of origin and spread to distant organs through invasion of the vascular system and/or the lymphatic system. Tumour vascularisation is widely accepted as a *bona fide* indicator of tumour growth, metastases and patient survival. In 1996, Peter Vermeulen *et al* (1996) published a first international consensus on the methodology and criteria of the evaluation of angiogenesis quantification in solid tumours and 5 years later, a second consensus report, in which new concepts and mechanisms of tumour vascularisation were integrated, appeared (Vermeulen *et al*, 2002). Both reports were aimed at improving the standardisation of angiogenesis quantification in order to allow intratumourous microvessel density to be applied as a prognostic indicator and, moreover, as a reliable predictor of the risk of malignant transformation of premalignant lesions and of response to cancer treatment. Contrary to angiogenesis, the *de novo* formation of lymphatic vessels or lymphangiogenesis and its role in promoting the metastatic spread of tumour cells has only recently become a focal point of cancer research with an increasing number of studies showing a relationship between patient survival and lymphatic density in different tumour types. In order to confirm the potential prognostic value of lymphangiogenesis in patients with cancer, a quantification method that is characterised by a low intra- and interobserver variability needs to be developed. In this first consensus report, we would like to provide an overview of current concepts of the lymphatic vasculature and its regulating factors and propose guidelines for the estimation of the ongoing lymphangiogenesis in solid human tumour sections.

## 

### Structural and molecular characteristics of the lymphatic vasculature

The vascular and lymphatic systems play complementary roles in tissue perfusion and subsequent extracellular fluid reabsorption. Lymphatic vessels comprise a complex open-ended capillary network that collect lymph from various organs and tissues. Lymphatic vessels are lined by a single layer of nonfenestrated endothelium that is attenuated over most of its surface, except in the perinuclear region which bulges into the lumen (Leak, 1976). Lymphatic endothelium abut an incomplete or absent basement membrane and has overlapping junctional complexes. Lymphatics are attached to the underlying matrix through anchoring filaments (Leak and Burke, 1968), which keep the vessel patent and therefore aid lymphatic flow even in areas with elevated hydrostatic pressure and these filaments may mediate outside-in signalling from the extracellular matrix akin to integrins. The complex anchoring filaments–focal adhesions may also control the permeability of lymphatic endothelium and finely adjust lymph formation to the physiological conditions of the extracellular matrix.

There are some differences in structure in different parts of the lymphatic system. Lymphatic vessels in tissues are absorbing capillaries with walls consisting solely of endothelium that drain into collecting vessels. Collecting lymphatic vessels have a thin circumferential extracellular coat and pericytes that reduce lymphatic fluid extravasation (Pepper and Skobe, 2003b). The transition between the absorbing and collecting vessels occurs gradually and so-called precollectors have been described which drain into prenodal collecting vessels with an irregular and tortuous course. The precollectors and collecting lymphatic vessels also have valves that enable uni-directional lymph flow (Swartz and Skobe, 2001).

Vascular and lymphatic endothelial cells share many similarities (Alitalo *et al*, 2005). Indeed, although initially thought to be restricted to blood vascular endothelial cells (BVECs), rod-like electrondense Weibel-Palade bodies containing Factor VIII-related antigen (von Willebrand factor) have been reported in lymphatic endothelial cells (LECs) (Di Nucci *et al*, 1996; Marchetti, 1996; Sacchi *et al*, 1999). Furthermore, 98% of genes were expressed at comparable levels by BVECs and LECs in culture (Petrova *et al*, 2002; Podgrabinska *et al*, 2002; Hirakawa *et al*, 2003) with the differences in those genes being involved in the regulation of lymphangiogenesis and lymphatic function (Sleeman *et al*, 2001) (vide infra). Nevertheless, akin to vascular endothelium, comparative studies suggest that different lymphatic endothelia have different phenotypes that are likely to mediate various biological activities (Garrafa *et al*, 2006).

### Molecular players of tumour lymphangiogenesis

Similar to angiogenesis, the growth of lymphatic vessels is regulated by a large number of growth factors (Table 1, Figure 1). Initially, members of the vascular endothelial growth factor (VEGF) family, VEGF-C and VEGF-D, were thought to be the only lymphangiogenic factors that stimulate lymphangiogenesis via activation of VEGFR-3 specifically expressed on normal LECs (Oh *et al*, 1997; Achen *et al*, 1998). The observation that a number of tumours expressing these two factors at low or undetectable levels that metastasize via the lymphatic system suggests that additional signalling systems probably exist (Cao, 2005a).

In xenographic and transgenic mouse tumour models, the overexpression of VEGF-A in tumours leads to lymphatic metastasis via intra- and peritumourous lymphatic vessels (Hirakawa *et al*, 2005; Bjorndahl *et al*, 2005b). It appears that VEGF-A indirectly induces lymphangiogenesis by recruiting VEGFR-1 expressing inflammatory cells including monocytes/macrophages and neutrophils that produce VEGF-C/-D because VEGFR-3 antagonists are able to block VEGF-A-induced lymphangiogenesis (Cursiefen *et al*, 2004). However, the direct effect of VEGF-A on lymphangiogenesis has also been reported since VEGFR-2 is expressed in LECs (Hong *et al*, 2004). Similar to VEGF-A, FGF-2 can indirectly induce lymphangiogenesis via the VEGF-C/-D/VEGFR-3 pathway by the recruitment of inflammatory cells, although a recent study also shows that FGF-2 may also directly stimulate the growth of LECs *in vitro* and lymphangiogenesis *in vivo* (Kubo *et al*, 2002; Chang *et al*, 2004; Shin *et al*, 2005). PDGF-BB was only recently described as a direct lymphangiogenic factor that promotes lymphatic metastasis (Cao *et al*, 2004). Both PDGF receptor alpha (PDGFR-a) and beta (PDGFR-b) are expressed on isolated LECs and all three prototypes of PDGFs, PDGF-AA, PDGF-BB and PDGF-CC, are able to induce lymphangiogenesis (Cao *et al*, 2004). Members of the angiopoietin (Ang-1 and -2), hepatocyte growth factor (HGF) and insulin-like growth factor (IGF-1 and IGF-2) family are newly reported direct lymphangiogenic factors (Gale *et al*, 2002; Morisada *et al*, 2005; Bjorndahl *et al*, 2005a; Cao, 2005b).

These known lymphangiogenic factors exhibit overlapping angiogenic activity on blood vessels. Thus, exposure of these growth factors to blood vessels and lymphatic vessels leads to simultaneous stimulation of angiogenesis and lymphangiogenesis (Cao, 2005b). However, under certain circumstances, both FGF-2 and VEGF-A have been reported to specifically induce only lymphangiogenesis and not angiogenesis (Chang *et al*, 2004; Bjorndahl *et al*, 2005b). The molecular mechanism underlying the differential effects of the same factor is currently unknown.

### Quantification of tumour lymphangiogenesis: prognostic/predictive value in oncology

In most cancers, lymph node (LN) metastasis is an important prognostic factor. However, LN status does not allow a solid prediction of prognosis for patients presenting with small tumours without LN involvement. Other reliable markers predictive of LN metastasis might improve prognostication and might be useful for therapeutic decision-making in these early cancers. Information about lymphatic invasion and the number of lymphatic vessels has been shown promising in this regard. In breast cancer, for example, the invasion of tumour cells into lymphatic vessels was shown to be predictive of LN involvement and a prognostic factor for overall and disease-free survival (Schoppmann *et al*, 2004; Lee *et al*, 2006). Indeed, peritumourous vascular invasion, especially lymphovascular invasion (LVI), has been included as a novel adverse prognostic factor in a series of guidelines and recommendations for postoperative adjuvant systemic therapies of early breast cancer developed by an International Consensus Panel during the St Gallen Conference, 2005 (Goldhirsch *et al*, 2005). The presence of peritumourous vascular invasion defined an intermediate risk for patients with node-negative breast disease, but its value in patients with node-positive breast disease was considered uncertain and insufficient at that time. Another clear example is early gastric cancer, in which the incidence of LN micrometastasis has been shown to be higher in patients with, than without LVI, indicating a close link between LVI and the initial stage of LN metastasis (Arigami *et al*, 2005). Lymphovascular invasion was found to be an adverse prognostic indicator in several studies of gastric cancer (Hyung *et al*, 2002; Kooby *et al*, 2003; Dicken *et al*, 2006). Other examples are node-negative bladder carcinoma (Lotan *et al*, 2005) and node-negative oesophageal carcinoma (Vazquez-Sequeiros *et al*, 2002) in which LVI was shown to be correlated with outcome. These data suggest that LVI may provide useful information for prognosis and clinical management in those patients who present with early tumours without LN involvement.

A correlation of lymphatic vessel density (LVD) detected by immunohistochemistry with an unfavourable prognosis has been observed in breast cancer, head and neck cancer, melanoma, cervical cancer, non-small-cell lung cancer, bladder cancer, colorectal cancer and gastric cancer. However, for the amount of lymphatic vessels in a solid tumour to be a reliable marker of prognosis, the quantification technique has to be characterised by a low intra- and interobserver variability. Results obtained at different institutes should be comparable in order to allow meta-analyses. Recently, the Programme for the Assessment of Clinical Cancer Tests Strategy group and a working group of a NCI-EORTC collaboration have reported guidelines for tumour marker studies with the objectives of facilitating the evaluation of the appropriateness and quality of study design, methods, analyses, and improving the ability to compare results across studies (McShane *et al*, 2005). In Table 2 we listed prognostic studies showing an association of LVD with the survival of patients with cancer and indicated how well the REporting Recommendations for tumour MARKer (REMARK) were followed as a tool for the reader. This consensus report aims to lower the methodological variability of lymphangiogenesis quantification in tumour tissue sections, bearing the REMARK guidelines for prognostic studies in mind.

## LYMPHATIC ENDOTHELIAL-SPECIFIC ANTIBODIES FOR IMMUNOHISTOCHEMISTRY

**Figure 2:**
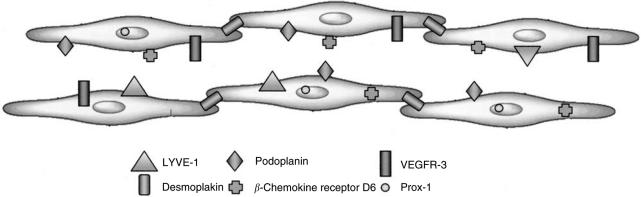
Representation of lymphangiogenic markers on lymphatic endothelial cells.

The selection of the optimal marker of the lymphatic endothelium is clearly a critical step in the assessment of LVD since false data arising from low specificity of the staining must be avoided. Major research efforts during these last years have lead to the discovery of a large spectrum of candidate lymphatic markers (Figure 2). The following paragraphs focus on markers for which antibodies are available and provide a discussion on the specificity of each marker for LECs.

### VEGFR-3

The vascular endothelial growth factor receptor 3 (VEGFR-3/Flt4) is a tyrosine kinase that is predominantly expressed in LECs in adult tissues (Kaipainen *et al*, 1995; Kukk *et al*, 1996). As VEGFR-3 expression has also been found in fenestrated capillaries of several organs including the bone marrow, splenic and hepatic sinusoids, kidney glomeruli and endocrine glands (Partanen *et al*, 1999) and in endothelial cells of the proliferating neovasculature in breast cancer (Valtola *et al*, 1999), this marker is not reliable for discriminating between lymphatic and blood vascular endothelium.

### Desmoplakin

The glycoprotein desmoplakin locates exclusively to the intracellular junctions between the endothelial cells of lymphatic vessels (Schmelz and Franke, 1993). Antibodies against desmoplakin have indicated specificity for lymphatic endothelium in human tongue (Ebata *et al*, 2001) but further studies are required to confirm the distinctive nature of desmoplakin staining in other tissue types.

### *β*-chemokine receptor D6

By using *in situ* binding assays it was shown that the *β*-chemokine receptor D6 is expressed on LECs in the skin (Hub and Rot, 1998). Monoclonal antibodies raised against the receptor specifically stained endothelial cells that were also stained with antipodoplanin antibodies (see below) and showed no immunoreactivity with endothelial cells lining the blood vessels (Nibbs *et al*, 2001). D6-immunoreactive lymphatic vessels were also abundant in mucosa and submucosa of small and large intestine and appendix, but were not observed in heart, kidney, liver, skeletal muscle, brain, cerebellum, pancreas, prostate and thyroid. This demonstrates the emerging heterogeneity of lymphatic endothelium, and it may be necessary to use specific markers depending on which tissue is being investigated.

### Prox-1

Another marker of the lymphatic endothelium is the homeodomain protein Prox-1, which is required for the regulation of lymphatic vascular development from pre-existing embryonic veins (Wigle and Oliver, 1999). Prox-1 expression has also been found in other cell types, including nonendothelial cells in the lens, heart, liver, pancreas and nervous system (Stacker *et al*, 2002). Antibodies against human Prox-1 to visualise lymphatic vessels in tumour sections have only been used in a limited number of studies (Agarwal *et al*, 2005; Van der Auwera *et al*, 2005). Although its nuclear localisation makes Prox-1 not the most ideal marker for quantifying lymphatic vessels microscopically, it could be a useful marker for double immunostaining with other markers such as podoplanin and LYVE-1.

### LYVE-1

LYVE-1 is an integral membrane glycoprotein that functions as a receptor for hyaluronan (GlcNAcb1–4GlcUAb1–3)_*n*_, a ubiquitous extracellular matrix glycosaminoglycan involved in cell migration and differentiation. The expression of LYVE-1 in endothelial cells of lymphatic vessels and LN sinuses and its absence from blood vessel endothelium was first demonstrated by immunohistochemical staining with polyclonal antibodies generated against recombinant human and murine LYVE-1 Fc fusion protein which showed characteristic staining of lymphatic vascular structures in skin, intestine and secondary lymphoid tissue (Banerji *et al*, 1999; Prevo *et al*, 2001). During embryogenesis, LYVE-1 is expressed in cardinal vein endothelium, just before budding of the primordial lymph sacs (E12.5 in the mouse), almost simultaneous with the expression of the lymphogenic transcription factor Prox-1 (Wigle and Oliver, 1999); expression then persists into adulthood in most afferent vessels and lymphatic sinuses, but is absent from thoracic duct. LYVE-1 is also abundant in discontinuous endothelia including human and mouse liver sinusoids and human spleen sinusoids but is absent from the ‘normal’ haemovasculature. Extensive analyses in many different laboratories have confirmed these findings and demonstrated that LYVE-1 is a reliable marker for distinguishing lymphatic vessels from blood vessels in a range of different human cancers (e.g. head and neck squamous cell carcinoma (Beasley *et al*, 2002; Maula *et al*, 2003), cutaneous melanoma (Dadras *et al*, 2003; Straume *et al*, 2003) and carcinomas of the thyroid (Hall *et al*, 2003), lung (Koukourakis *et al*, 2005), pancreas (Von Marschall *et al*, 2005), breast (Williams *et al*, 2003; Bono *et al*, 2004), cervix (Van Trappen *et al*, 2003) and prostate (Trojan *et al*, 2004)), as well as normal tissues in both adult and foetus. Nevertheless, the observation that expression of LYVE-1 can be downmodulated in some tissues, for example, in response to inflammation (Johnson L and Jackson DG, unpublished), and is absent in some tumour-associated lymphatics (Rubbia-Brandt *et al*, 2004; Stessels *et al*, 2004; Van der Auwera *et al*, 2004) underlines the importance of utilising multiple markers (e.g. LYVE-1/podoplanin, LYVE-1/Prox-1, etc.) to characterise lymphatic vessels in comprehensive studies of lymphangiogenesis. Besides lymphatic and sinusoidal vessel endothelium, LYVE-1 is also expressed in some macrophage-like cells present in inflamed tissue and in tumour infiltrates. The special significance of these findings has been revealed in recent studies of lymphangiogenesis occurring during corneal neovascularisation (Maruyama *et al*, 2005) and in transplanted kidney rejection (Kerjaschki *et al*, 2006) where LYVE-1^+^/CD68^+^ cells were shown to be incorporated into newly dividing lymphatic vessels. These findings indicate the fascinating possibility that LYVE-1^+^ macrophage-like cells may represent bone marrow derived progenitors with the capacity to differentiate towards lymphatic vessel endothelium as well as regulators of VEGF-C induced lymphoproliferation (vide infra).

### Podoplanin

Podoplanin is a ∼38-kd surface glycoprotein that is expressed in osteoblastic cells, lung alveolar type I cells and kidney podocytes (Wetterwald *et al*, 1996; Breiteneder-Geleff *et al*, 1997). The specificity of podoplanin expression on lymphatic but not blood vascular endothelium has been demonstrated in the skin (Breiteneder-Geleff *et al*, 1999). However, podoplanin appears to be only present in small lymphatic vessels and not in larger ones that have smooth-muscle cells (Stacker *et al*, 2002). So far, there is no evidence of podoplanin expression in BVECs (Stacker *et al*, 2002), suggesting that it can be considered as a reliable marker of the lymphatic endothelium. Recently, it was indicated by Schacht *et al* (2005) that the commercially available monoclonal D2–40 antibody specifically recognises human podoplanin. The antibody has been shown to be a highly selective marker of lymphatic endothelium in sections of both frozen and formalin-fixed paraffin-embedded normal and neoplastic tissues (Kahn *et al*, 2002a) and has been proven valuable in detecting lymphatic invasion in various malignant neoplasms (Kahn and Marks, 2002b). In a direct comparison of the D2–40 antibody and an antibody against podoplanin on paraffin sections of a series of head and neck squamous cell carcinomas, both antibodies were shown to have extremely high specificity (99.7 and 98.8% for podoplanin and D2–40) and sensitivity (92.6 and 97.3% for podoplanin and D2–40) for lymphatic endothelium (Evangelou *et al*, 2005).

A comparative study of antibodies directed at LYVE-1, podoplanin, Prox-1 and the D2–40 antibody on serial sections of breast carcinomas indicated that significantly more intratumourous lymphatic vessels stained with D2–40 (Van der Auwera *et al*, 2005), thus demonstrating that this marker is highly sensitive for lymphatic endothelium. Besides being reactive with lymphatic vessels, D2–40-staining has also been observed in basal epithelial cell layers of the epidermis (Niakosari *et al*, 2005) and of human breast and prostate gland (Agarwal *et al*, 2005; Zeng *et al*, 2005).

Just as tumour vasculature has a markedly different phenotype from normal vessels, so it is highly likely that tumour lymphatic vessels will differ from normal and gene array studies on lymphatic endothelium isolated from tumours will be of major interest to help develop new markers relevant to tumour therapy and outcome. Fiedler *et al* (2006) very recently reported that the CD34 protein, a recognised vascular endothelial marker, is selectively expressed in tumour-associated LECs and not in resting organ LECs. The expression of CD34 by tumour-associated LECs was identified in colon cancer, breast cancer, lung cancer and melanoma. These findings underline the importance of CD34 as an activation antigen of human LECs and as a potential diagnostic and prognostic tumour marker.

## METHODOLOGY OF LYMPHANGIOGENESIS QUANTIFICATION IN SOLID TUMOURS BY HISTOMORPHOMETRY

### Lymphatic vessel density

By analogy with angiogenesis, tumour-associated LVD is most frequently assessed by counting the number of immunostained vessels in tumour sections, as defined by Weidner *et al* (1991) in 1991. Microvessel density (MVD) is determined in vascular ‘hot spots’ or areas giving the impression at low magnification of containing numerous microvessels. Vascular ‘hot spots’ are thought to represent localised areas of biological importance since they originate from tumour cell clones with the highest angiogenic potential which will predominantly enter the circulation and give rise to vascularised metastases. Localised changes in oxygen tension are indeed a strong angiogenic drive. The reproducibility of the assignment of these ‘hot spots’ is a critical variable in the analysis of MVD and the success of finding the relevant ‘hot spot’ depends on the training and experience of the investigator (Vermeulen *et al*, 2002). The methodology of counting the number of microvessel entities in regions with an elevated vascular density has been adapted for the assessment of LVD, although this is based on the assumption that a functional increase in lymphatic vessels occurs in ‘hot spots’. Since data on the association of lymphangiogenesis with hypoxia are still contradictory, the relevance of counting lymphatic vessels in ‘hot spots’, as opposed to an overall lymphatic vessel count, has been questioned (Shields *et al*, 2004).

The number of lymphatic vessels in a microscopic field is the net result of previous phases of tumour lymphangiogenesis and of lymphatic vessel remodelling or regression, which implicates that the measurement of LVD is not necessarily a reflection of the ongoing tumour lymphangiogenesis. Nevertheless, several studies in different cancer types have found a correlation of LVD with lymphangiogenic factor expression, and with the occurrence of lymphatic metastases and survival, suggesting that LVD contains important information on the degree of tumour lymphatic vasculature.

There is still a considerable debate about the role of intratumourous *vs* peritumourous lymphatic vessels in the pathology of primary human tumours. Several studies have shown that the density of lymphatic vessels located immediately adjacent to the tumour is associated with the presence of LN metastases (Dadras *et al*, 2003; Bono *et al*, 2004; Franchi *et al*, 2004; Gombos *et al*, 2005; Hachisuka *et al*, 2005; Kyzas *et al*, 2005; Mou *et al*, 2005; Renyi-Vamos *et al*, 2005; Zeng *et al*, 2005; Massi *et al*, 2006; Roma *et al*, 2006). Moreover, in a retrospective prognostic study, Dadras *et al* (2003) found that the size of peritumourous lymphatic vessels was the most significant independent factor that correlates with LN metastasis in human malignant melanomas. However, other studies show that intratumourous and not peritumourous lymphatic vessels are vital for lymphatic metastasis (Beasley *et al*, 2002; Hall *et al*, 2003; Maula *et al*, 2003; Audet *et al*, 2005; Kuroyama *et al*, 2005; Kyzas *et al*, 2005; Massi *et al*, 2006).

The infiltration of lymphatic vessels into the tumour may have a passive role in cancer metastasis by creating an increased opportunity for metastatic tumour cells to leave the primary tumour site but might also establish a paracrine signalling pathway for tumour cell growth and invasion through the release of specific growth factors or chemokines (Cassella and Skobe, 2002). Michaela Skobe *et al* have shown that lymphatic capillaries activated by factors produced by tumours, such as VEGF-C, promote tumour cell invasion by increasing tumour cell transendothelial migration through the expression of the CC-type chemokine ligand 1 on LECs and its receptor CC-type chemokine receptor 8 on tumour cells (Alitalo *et al*, 2004). Moreover, secondary lymphoid chemokine is constitutively produced by LECs in the skin (Saeki *et al*, 1999) and other organs (Gunn *et al*, 1998) and was found to attract dendritic cells to the lymphatic vessels by interaction with its primary receptor CCR7. This chemokine receptor is highly expressed in human breast cancer cells, malignant breast tumours and metastases, triggering actin polymerisation, pseudopodia formation, and the directional migration and invasion of these cells (Muller *et al*, 2001).

### Chalkley counting

Although tumour-associated lymphangiogenesis has mainly been assessed by counting the number of immunostained lymphatic vessels, other techniques, such as Chalkley point overlap morphometry, are available. This method involves the use of an eyepiece graticule containing 25 randomly positioned dots, which is rotated so that the maximum number of points is on or within the vessels of the vascular ‘hot spot’. Thus, instead of counting the individual microvessel, the overlaying dots are counted. Hall *et al* (2003) investigated the relationship between LVD, determined by Chalkley counting, and clinical and pathological variables in patients with well-differentiated papillary thyroid carcinoma. In a multivariate analysis, the Chalkley score was found to be significantly associated with the presence of nodal metastases at presentation. A similar association has also been shown in head and neck cancer (Audet *et al*, 2005).

The Chalkley count is a reflection of the relative area taken by the lymphatic vasculature and offers a suitable alternative for LVD assessment according to Weidner's guidelines. As no decisions have to be made on whether adjacent stained structures are separate microvessel or not, Chalkley point counting should be a more objective approach. The most observer-dependent step though still remains, that is, the selection of the vascular ‘hot spot’.

### Lymphatic endothelial cell proliferation (LECP)

LECP is measured by a double immunostaining of tumour sections with antibodies directed at a LEC marker (antipodoplanin or anti-LYVE-1) and a marker of proliferating cells (anti-Ki67 or anti-PCNA). Lymphatic vessels containing proliferating nuclei have been observed in breast cancer (Van der Auwera *et al*, 2005), endometrial cancer (Koukourakis *et al*, 2005), head and neck cancer (Beasley *et al*, 2002) and melanoma (Dadras *et al*, 2003; Straume *et al*, 2003). This suggests the presence of active intratumourous lymphangiogenesis, at least in some tumour types. In addition to the sprouting of lymphatic vessels, the enlargement of lymphatic vessels is also accompanied by the proliferation of LECs. It has been reported that both lymphangiogenesis and lymphatic hyperplasia play a role in tumour dissemination (Skobe *et al*, 2001; Stacker *et al*, 2001; He *et al*, 2005). In a VEGF-C overexpressing animal model, a tumour-induced increase in the diameter of collecting lymphatic vessels was associated with an enhanced passage of clusters of tumour cells to the sentinel LNs (He *et al*, 2005). Increased lymphatic vessel perimeters and areas were also found to be correlated with the occurrence of lymphatic metastasis in some human tumours (Nathanson *et al*, 1997; Dadras *et al*, 2003; Franchi *et al*, 2004; Van der Auwera *et al*, 2005; Liang *et al*, 2006; Massi *et al*, 2006).

### Computerised image analysis systems

The major drawbacks of the visual MVD counting method are its inherent subjectivity and the difficulty of standardisation between laboratories. In contrast, image cytometry is more objective and reproducible and moreover, many image cytometry software packages allow additional information on vessel luminal area and vessel luminal perimeter. However, the widespread application of image cytometry is hampered by the need for specialised equipment to perform the analyses. Another limitation of this method is the possibility of confounding signals of nonendothelial structures in the stromal compartment. Choi *et al* (2005) performed a direct comparison of visual and image cytometric lymphatic vessel density counting on D2–40-immunostained sections of invasive breast carcinomas. They used an automated scanning microscope and an automated image analysis application that identified stained ring-like structures based on colour and morphometry in areas marked during direct microscopic microvessel counting. D2–40 microvessel densities determined by direct microscopy and image cytometry were significantly correlated. However, only the visual D2–40 data were associated with LN status and VEGF-family gene expression.

## SURROGATE MARKERS OF TUMOUR-ASSOCIATED LYMPHANGIOGENESIS

### Histopathological markers

#### Fibrotic focus

Similar to angiogenesis, the fibrotic focus and the growth pattern might be considered as surrogate histopathological markers for tumour-associated lymphangiogenesis (Vermeulen *et al*, 2002). A fibrotic focus is defined as a fibrosclerotic scar-like area replacing necrosis in the centre of a carcinoma. The presence of a fibrotic focus in breast cancer is considered to be a surrogate marker of hypoxia-driven angiogenesis as it was shown to predict for higher MVD and for a higher fraction of proliferating endothelial cells (Jitsuiki *et al*, 1999; Colpaert *et al*, 2003a). Similarly, its presence in breast cancer is associated with a higher LECP but not with a higher LVD (Van der Auwera *et al*, 2005).

#### Tumour growth pattern

It has been previously shown that different growth patterns in primary breast cancer reflect differences in angiogenesis. In the infiltrative growth pattern, the carcinoma cells invade between pre-existing structures without a significant disturbance of the tissue architecture. Expansively growing breast tumours form a well-circumscribed nodule consisting of carcinoma cells and desmoplastic connective tissue. The endothelial cell proliferation fraction and the Chalkley count were highest in the expansive growth pattern (Colpaert *et al*, 2003b; Van den Eynden *et al*, 2005). Recently, it became clear that the growth pattern is also a histological surrogate marker of lymphangiogenesis (Van der Auwera *et al*, 2005). LECP, both in the tumour parenchyma and at the tumour periphery, was significantly higher in the expansive growth pattern compared with the infiltrative growth pattern. In addition, the intratumourous LVD was highest in the infiltrative growth pattern. An association between the growth pattern and the presence of lymphangiogenesis has also been observed in human non-small-cell lung cancer (Renyi-Vamos *et al*, 2005).

#### Tumour levels of lymphangiogenic growth factors

The expression, in various human cancers, of lymphangiogenic factors such as VEGF-C and VEGF-D, is closely related to tumour-induced lymphatic dilatation or lymphangiogenesis (less frequent) and thereby to LN metastasis (Pepper *et al*, 2003a).

In breast cancer, increased expressions of VEGF-C and VEGF-D in the tumour cells, both on the mRNA and protein level, are known to be associated with high LVD, lymphatic invasion and LN metastasis (Nakamura *et al*, 2003; Choi *et al*, 2005; Nakamura *et al*, 2005; Huang *et al*, 2006; Li *et al*, 2006). Straume *et al* (2003) have compared LVD, evaluated by counting the number of LYVE-1-positive vessels in hot spots, with the protein expression of several (lymph)angiogenic growth factors in cutaneous melanoma specimens. Among the factors tested (VEGF-A, VEGF-C, VEGFR-1, VEGFR-2, VEGFR-3, FGF-2, ephrin-A1/2, interleukin-8 and thrombospondin-1), only the expression of FGF-2 was significantly associated with increased LVD. Furthermore, evidence for the existence of an association between VEGF-C or VEGF-D expressions and the number of tumour lymphatic vessels has also been provided for colorectal cancer (Ohno *et al*, 2003; Jia *et al*, 2004; Wang *et al*, 2005), gastric cancer (Yonemura *et al*, 2001; Onogawa *et al*, 2005; Shida *et al*, 2005; Juttner *et al*, 2006), thyroid cancer (Yasuoka *et al*, 2005), early-stage squamous cell cancer of the urerine cervix (Gombos *et al*, 2005) and pancreatic cancer (Sipos *et al*, 2004). In non-small-cell lung cancer, however, the existence of an association between VEGF-C expression and LVD remains contradictory (Lu *et al*, 2005; Renyi-Vamos *et al*, 2005; Takanami, 2006).

### Other markers

The main disadvantage of the histological surrogate markers of lymphangiogenesis is the inherent interobserver variability. A more objective approach is the quantification of circulating levels of lymphangiogenic growth factors, such as VEGF-C and VEGF-D, and of circulating lymphatic endothelial progenitor cells.

#### Circulating levels of lymphangiogenic growth factors

From a practical point of view, the detection of circulating levels of VEGF-C and VEGF-D protein/antigen in preoperative blood samples might be a useful indicator of advanced disease.

In some cancers, circulating lymphangiogenic factors are increased compared to healthy individuals or patients with benign tumours, for example, VEGF-C in lung non-small-cell carcinoma (Tamura and Ohta, 2003). However, in ovarian carcinoma, breast carcinoma, cervix adenocarcinoma and head and neck carcinoma this seems not to be the case (Table 3). Furthermore, in colorectal cancer, VEGF-C is increased in cancer patients but not VEGF-D (George *et al*, 2001; Duff *et al*, 2005). In lung non-small-cell carcinoma, cervical squamous cell carcinoma and colorectal cancer, a higher preoperative circulating VEGF-C level strongly correlated with LN metastasis and also lymphatic vessel invasion (the latter only in lung non-small-cell carcinoma). Concerning VEGF-D, plasma levels are increased in patients with angiosarcoma compared to healthy controls, and in one study on prostate carcinoma, a correlation between VEGF-D levels and LN metastasis was found (Kaushal *et al*, 2005).

The above results should be seen in context, since there are only a few studies on circulating VEGF-C and VEGF-D levels compared to the expression of their mRNA or protein levels in cancer.

#### Lymphatic endothelial progenitor cells (LEPCs)

Bone marrow-derived circulating endothelial precursor cells contribute to newly blood vessel formation both under physiological and pathological conditions. The measurement of circulating endothelial cells in peripheral blood of patients with cancer has been integrated in clinical studies exploring the efficacy of antiangiogenic therapies. Evidence for the existence of LEPCs has only recently been found. Salven *et al* (2003) identified a subset of CD34+ cells that coexpress the stem/precursor cell marker CD133 and VEGFR-3. These cells are functionally nonadherent endothelial precursor cells that can differentiate into mature adherent VEGFR-3+ endothelial cells in the presence of vascular growth factors. In a corneal lymphangiogenesis model of irradiated mice reconstituted with donor BM cells, both CD34+/VEGFR-2+ cells and CD34+/VEGFR-3+ cells were found to be incorporated into the newly formed lymphatic vessels (Religa *et al*, 2005). A second population of candidate LECPs has now been identified. A subpopulation of human circulating CD14+ monocytes was shown to also express VEGFR-3 on its surface and could be stimulated *in vitro* to express VEGF-C as well as the LEC marker podoplanin (Schoppmann *et al*, 2002; Kerjaschki *et al*, 2006). It was speculated that these cells participate in inflammation-associated *de novo* lymphangiogenesis in nephrectomy specimens of rejected kidney transplants (Kerjaschki *et al*, 2006). Maruyama *et al* (2005) have provided direct evidence of the incorporation of transdifferentiated monocytes-macrophages into growing lymphatic vessels. In a mouse corneal transplantation model, macrophages could transdifferentiate into LECs by forming cell aggregates and vesicles that integrate into an existing lymphatic vessel. Moreover, *in vitro* experiments demonstrated that CD11b+ macrophages were capable of forming tube-like structures that expressed markers of lymphatic endothelium such as LYVE-1 and podoplanin. These data indicate a novel role for macrophages in lymphangiogenesis. Gene expression profiling studies are necessary for characterisation of LECPs in peripheral blood and will improve our understanding of lymphatic endothelial function in cancer.

## RECOMMENDED METHODOLOGY

So far, studies on the importance of lymphangiogenesis for tumour growth have yielded inconsistent conclusions and this is mostly due to differences in the applied methodology and the lack of standardisation. In Table 4, a proposition for the standardisation of the immunohistochemical method for lymphangiogenesis assessment is given.

Methods of lymphangiogenesis quantification in solid tumours rely upon the use of markers that allow an accurate discrimination between lymphatic vessels and blood vessels in histological tissue sections. At present the most reliable marker is likely to be podoplanin, which is recognised by the monoclonal D2–40 antibody with a high specificity and sensitivity. Although LYVE-1 has been proven valuable for distinguishing between lymphatic vessels and blood vessels in histological tissue sections, it has been reported that LYVE-1 expression in tumour lymphatic vessels can be downmodulated, for example, in breast cancer (Stessels *et al*, 2004; Van der Auwera *et al*, 2004). None of the proposed markers fulfils the criteria of an ideal lymphatic vessel marker, which should be exclusively found on all types of LECs in all pathological conditions. Therefore, the use of multiple immunohistochemical stains on serial sections of random subgroups of cases is recommended to confirm the actual staining of lymphatic vessels. The best combination of markers of the lymphatic endothelium could vary on the tissue type. As more insights on the molecular pathways of lymphatic differentiation emerge, novel potential markers of the lymphatic endothelium might be identified.

The quantification of LVD has been proven valuable for the risk assessment of regional LN involvement in patients with cancer. By using the Chalkley point overlap morphometric technique the observer-dependent step of measuring LVD can be abolished since the Chalkley count is a relative area estimate rather than a true vessel count. However, the method that most likely reflects the ongoing tumour lymphangiogenesis would be the analysis of proliferating LECs, which can be assessed by a double immunostain with podoplanin to stain lymphatic vessels, together with Ki-67 to stain proliferating cells.

As it seems that the patterns of lymphangiogenesis vary among malignancies lymphangiogenesis should be evaluated both intratumourous and at the tumour periphery.

## SUMMARY AND FUTURE DIRECTIONS

For every major type of cancer LN involvement is strongly associated with poor survival and usually is one of the major factors associated with poor prognosis. Whether this is the mechanism for poor prognosis or a marker for aggressive underlying molecular pathway is difficult to determine clinically. The pathway of metastasis via lymphatic vessels, regional LNs and then into the systemic circulation is an accepted pathway of metastasis, although in recent years the emphasis has switched to the importance of angiogenesis and direct systemic spread. Nevertheless, it is not possible to quantify the relative contribution to spread currently between these different routes and the very strong association of LN involvement with outcome is a key factor in the staging of all cancers. Understanding such an important prognostic factor, the mechanisms regulating it and how it might be related to prognosis are important issues regardless of any possible therapeutic approach.

Understanding the mechanisms by which lymphangiogenesis occurs or tumour cells migrate to lymphatic vessels, or indeed understanding how new lymphatic vessels are generated from other cell types such as macrophages or circulating progenitor cells is all highly relevant to potential mechanisms of growth of metastases. VEGF-A-overexpressing primary tumours have been shown to induce sentinel LN lymphangiogenesis before metastasising (Hirakawa *et al*, 2005) and also in human cancer lymphangiogenesis appears to occur in secondary sites, for example, in LN metastases of breast cancer (Van den Eynden *et al*, 2006). This might suggest that primary tumours begin preparing their future metastatic site by inducing the growth of new lymphatic vessels.

Lymphangiogenesis is a complex process that is regulated by multiple factors that are produced by various cell types. Some of the originally angiogenic signalling molecules, such as VEGF-A and FGF-2, have been implicated in the control of lymphatic vessel growth as well, indicating a close link between angiogenesis and lymphangiogenesis. Novel insights into the interrelationship between both processes will lead to a better understanding of the mechanisms of growth of metastases and will have important implications for cancer therapy. Many potent antiangiogenic compounds that can be used in anticancer therapy have been identified and are currently being investigated in clinical trials. It is not clear whether these antiangiogenic agents also affect lymphangiogenesis and hence, biopsy studies should also stain for effects on lymphatic vessels to dissect their possible role in response to therapy.

The search for specific lymphangiogenesis inhibitors has led to the identification of a number of potential antilymphangiogenic compounds that have been shown to suppress metastasis of tumours to regional LNs in experimental animal models. These include antibodies that either block the activity of the ligands VEGF-C and VEGF-D directly by binding to the ligand or by preventing the interaction with VEGFR-3 and soluble dimeric fusion proteins containing the extracellular ligand binding site of VEGFR-3 (Banerji *et al*, 1999; Thiele and Sleeman, 2006). However, many questions concerning their potential therapeutic role in the management of human cancer are raised. Although the point could be made that when patients first present they already have lymphatic metastasis or not, and these will be treated surgically or with other means, it would be highly desirable to prevent metastasis in patients who are at increased risk of second primaries. A clear example is breast cancer with second cancers either in the treated breast or the opposite breast and reducing the incidence of new primaries, but also reducing the instance of secondary deposits from the new primaries is a key therapeutic aim.

Understanding the mechanisms of lymphangiogenesis could be helpful in the adjuvant situation of managing common cancers with treatment aimed at both stopping proliferation and recurrence of primary tumours and their regional metastases.

## Figures and Tables

**Figure 1:**
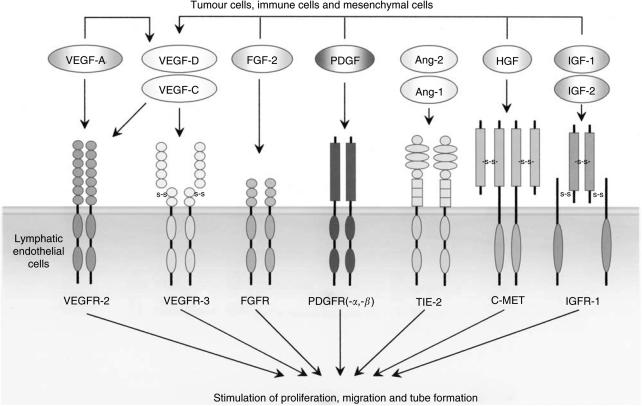
Lymphangiogenic growth factors and their receptors.

**Table 1:** Molecular players of lymphangiogenesis

**Gene**	**Involvement in lymphangiogenesis**	**Reference**
*VEGF-C*	Essential for sprouting the first lymphatic vessel from Prox-1-positive endothelial cells of veins	Karkkainen *et al* (2004)
	Overexpression in mouse tumour models promotes the growth of intratumourous lymphatic vessels and metastasis to regional lymph nodes	Skobe *et al* (2001), Karpanen *et al* (2001)
		
*VEGF-D*	Overexpression in mouse tumour models induces the formation of lymphatic vessels within the tumour and leads to spread of the tumour to lymph nodes	Stacker *et al* (2001)
		
*VEGFR-3*	Plays an important role in the development of the lymphatic vasculature	Karkkainen *et al* (2000)
	Induces proliferation of cultured LECs	Mäkinen *et al* (2001)
	Induces lymphangiogenesis in transgenic mice	Veikkola *et al* (2001)
		
*VEGF-A*	Overexpression in mouse tumour models induces the growth of peritumourous lymphatic vessels and leads to lymphatic metastasis	Bjorndahl *et al* (2005b), Hirakawa *et al* (2005)
	VEGFR-2 is expressed in LECs	Hong *et al* (2004)
	Can induce lymphangiogenesis indirectly by recruiting VEGFR-1 expressing inflammatory cells including monocytes/macrophages and neutrophils	Cursiefen *et al* (2004)
		
*FGF-2*	Stimulates proliferation, migration and tube formation of cultured LECs	Chang *et al* (2004), Shin *et al* (2005)
	Induces sprouting of lymphatic vessels in a mouse corneal model can induce lymphangiogenesis indirectly by recruiting inflammatory cells	Chang *et al* (2004), Kubo *et al* (2002)
		
*PDGF-BB*	Stimulates cell motility of cultured LECs	Cao *et al* (2004)
	Isolated LECs express both PDGFR-alpha and beta	
	Overexpression in a mouse tumour model stimulates the growth of intratumourous lymphatic vessels and lymphatic metastasis	
		
*Ang-2*	Ang-2-knockout mice show disorganised and hypoplastic dermal and intestinal lymphatic capillaries	Gale *et al* (2002)
		
*Ang-1*	Restores lymphatic defects of Ang-2-knock-out mice	Gale *et al* (2002)
	Promotes LYVE-1-positive lymphatic vessel formation in murine cornea	Morisada *et al* (2005)
		
*HGF*	Stimulates proliferation, migration and tube formation of cultured LECs	Kajiya *et al* (2005)
	Induces sprouting and growth of new LYVE-1 expressing lymphatic vessels in mice corneal and tumour models	Cao *et al* (2006), Jiang *et al* (2005)
		
*IGF-1*	Stimulates proliferation and migration of cultured LECs	Bjorndahl *et al* (2005a)
	IGFR-1 is present in lymphatic endothelium	
	Induces growth of new LYVE-1 expressing lymphatic vessels in murine cornea	
		
*IGF-2*	Stimulates proliferation and migration of cultured LECs	Bjorndahl *et al* (2005a)
	IGFR-1 and -2 are present in lymphatic endothelium	
	Induces growth of new LYVE-1 expressing lymphatic vessels in murine cornea	

**Table 2:**
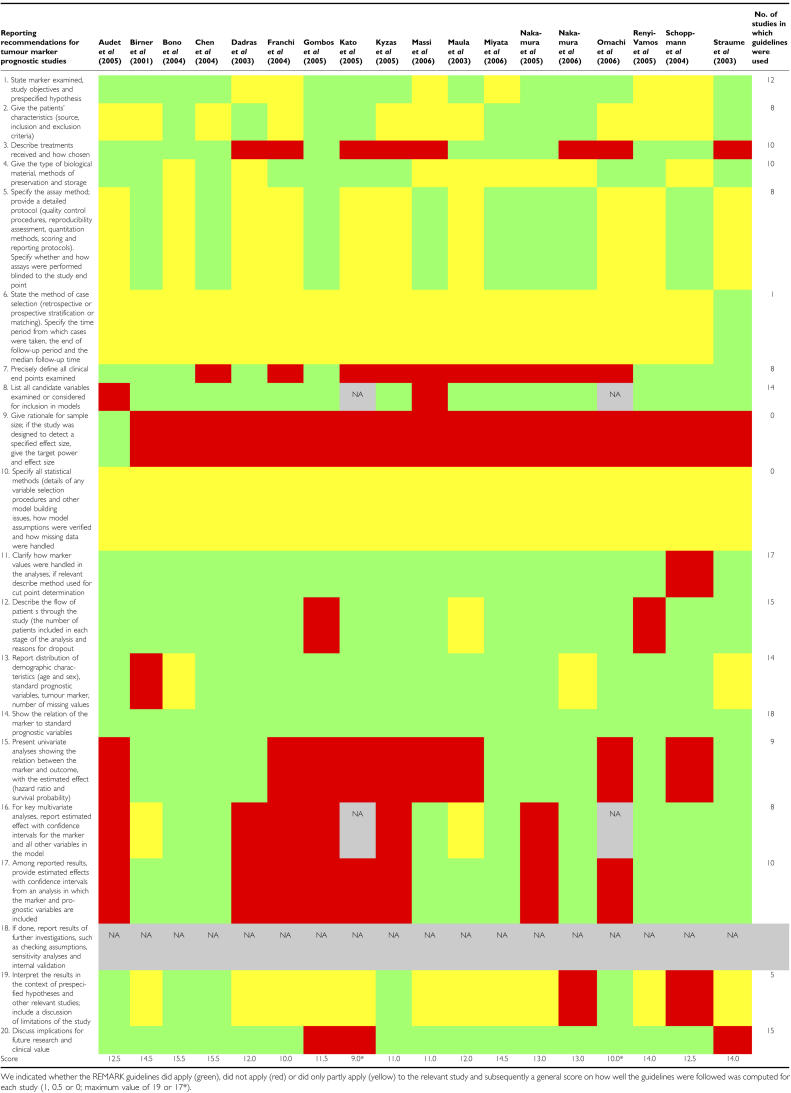
List of studies on the prognostic value of lymphangiogenesis in solid human tumours

**Table 3:** Studies on circulating VEGF-C and VEGF-D levels in patients with cancer

**Tumour**	**Markers (source)**	**Comments**	**Reference**
*Colorectal ca.*	VEGF-C (plasma)	Increased in cancer patient compared to control	Duff *et al* (2003)
		Significantly higher in advanced Dukes C and D (LN+) compared to Dukes A and B (LN−)	
	VEGF-C (plasma)	Decreased expression in cancer patient compare to control	Duff *et al* (2005)
	VEGF-D (serum)	No difference between cancer and control	George *et al* (2001)
	VEGF-D (plasma)	No difference between cancer and control	Duff *et al* (2005)
*Non-small cell lung ca.*	VEGF-C (serum)	Increased in carcinoma compared to benign lesions and control individuals	Tamura and Ohta (2003)
		Correlation with pathologic stage, LN metastasis and lymphatic vessel invasion	
	VEGF-C (serum)	Correlation with pathologic stage, LN metastasis and lymphatic vessel invasion	Tamura *et al* (2004a, 2004b)
*Cervical ca.*	VEGF-C (serum)	Increased in squamous cervical cancer compared to controls	Mitsuhashi *et al* (2005)
		Correlation with FIGO stage, tumour size and recurrence but not with LN metastasis	
		No increase in cervical adenocarcinoma compared to controls	
	VEGF-C (serum)	Increased in cervical cancer compared to controls	Mathur *et al* (2005)
*Prostate ca.*	VEGF-D (plasma)	Increased in early stage (LN neg) compare to late stage (LN or bone) metastasis	Kaushal *et al* (2005)
*Angiosarcoma*	VEGF-D (serum)	Increased compared to controls	Amo *et al* (2004)
*Ovarian ca.*	VEGF-C (serum)	Not increased compared to controls	Mathur *et al* (2005)
*Breast ca.*	VEGF-D (plasma)	Not increased compared to controls	Hoar *et al* (2004)
*Head and neck ca.*	VEGF-C (plasma)	Not increased compared to controls	Strauss *et al* (2005)

**Table 4:** Proposed standard method for the assessment of lymphangiogenesis

**Methodological aspect**	**Proposed standard**	**Advantage**
1. Immunostaining	Double immunostain with the D2-40 monoclonal antibody and the anti-Ki-67 monoclonal antibody	Highly specific and sensitive marker of the lymphatic endothelium
2. Selection of the quantification fields	Manual vascular hot spot selection at low magnification (e.g. × 10) – in viable tumour tissue and adjacent (e.g. within diameter of one field at × 200 magnification) stromal tissue	All highly vascular areas can be detected
3. Quantification of lymphatic vessels	Chalkley point graticule method	Exclusion of the subjective step of identifying individual lymphatic vessels in an endothelial cell cluster
4. Quantification of LEC proliferation	Counting of the number of proliferating LECs *vs* nonproliferating LECs	Reflection of the ongoing lymphangiogenesis
5. Number of observers	Sequential assessment by two investigators	More practical in a clinical setting than co-observation
